# Kinetics of Mosquito-Injected *Plasmodium* Sporozoites in Mice: Fewer Sporozoites Are Injected into Sporozoite-Immunized Mice

**DOI:** 10.1371/journal.ppat.1000399

**Published:** 2009-04-24

**Authors:** Chahnaz Kebaier, Tatiana Voza, Jerome Vanderberg

**Affiliations:** Department of Medical Parasitology, New York University School of Medicine, New York, New York, United States of America; Faculdade de Medicina da Universidade de Lisboa, Portugal

## Abstract

Malaria is initiated when the mosquito introduces sporozoites into the skin of a mammalian host. To successfully continue the infection, sporozoites must invade blood vessels in the dermis and be transported to the liver. A significant number of sporozoites, however, may enter lymphatic vessels in the skin or remain in the skin long after the mosquito bite. We have used fluorescence microscopy of *Plasmodium berghei* sporozoites expressing a fluorescent protein to evaluate the kinetics of sporozoite disappearance from the skin. Sporozoites injected into immunized mice were rapidly immobilized, did not appear to invade dermal blood vessels and became morphologically degraded within several hours. Strikingly, mosquitoes introduced significantly fewer sporozoites into immunized than into non-immunized mice, presumably by formation of an immune complex between soluble sporozoite antigens in the mosquito saliva and homologous host antibodies at the proboscis tip. These results indicate that protective antibodies directed against sporozoites may function both by reducing the numbers of sporozoites injected into immunized hosts and by inhibiting the movement of injected sporozoites into dermal blood vessels.

## Author Summary

Malaria is initiated by a mosquito injecting malaria sporozoites into the skin. To successfully continue the infection, sporozoites must then invade blood vessels in skin for transportation to the liver. However, the majority of these injected sporozoites are unable to reach the blood. The numbers of sporozoites that successfully invade the blood may influence the characteristics of the subsequent clinical malaria infection. We studied this by microscopy with fluorescent sporozoites of the rodent malaria parasite *Plasmodium berghei* injected into mice by mosquitoes. Sporozoites introduced into mice that have been immunized against sporozoites become immobilized and cannot reach the blood; those that remain at the bite site become degraded within several hours. Strikingly, mosquitoes introduce significantly fewer sporozoites into skin of immunized mice. These findings indicate that antibodies directed against sporozoites seem to function both by reducing the numbers of sporozoites injected into immunized hosts in the first place and then by inhibiting the movement of the injected sporozoites into the bloodstream.

## Introduction

It is now widely accepted that most if not all malaria sporozoites are deposited by mosquitoes into the avascular tissues of their mammalian hosts, from where their motility allows them to enter blood vessels for their subsequent journey to the liver and invasion of hepatocytes [1]–[6]. An early study [7] had shown rapid transfer of mosquito-injected sporozoites into the blood but failed to prove direct inoculation into the bloodstream, concluding instead that sporozoites were inoculated “into the tissues or directly into the blood vessels”. In our initial study, which we did by allowing *Plasmodium berghei*-infected mosquitoes to feed on mice and then extirpating the feeding site at various times post-feeding, we concluded that the first wave of sporozoites entered the blood circulation at around 15 min after feeding [1]. However, our approach did not allow us to determine the subsequent rate at which sporozoites continued to leave the skin or whether significant numbers of sporozoites remained in the skin for longer periods of time. We reported subsequently “that in addition to these early emigrants from the skin, substantially more sporozoites take a significantly longer time to enter the blood” and that “the relatively long time during which many sporozoites continue to migrate within the skin (for at least 30 min) was striking” [3]. This was confirmed and extended with *P. berghei* [5] and still later with *P. yoelii* [6], this latter study reporting that sporozoites inoculated by the mosquito are released from the skin into the blood circulation in a trickle extending for hours after the mosquito bite.

We decided to examine sporozoite deposition and the kinetics of sporozoite disappearance from tissues on which mosquitoes had fed, both in immunologically naïve mice and in mice that had been immunized against sporozoites. We had previously counted numbers of sporozoites injected into ear pinnae and abdominal tissue by direct fluorescence microscopy quantification of sporozoites expressing red fluorescent protein [8]. For the current study, we used the same procedure to count numbers of sporozoites in biopsy specimens taken at various times after mosquito injection into control and immunized mice to establish the kinetics of these sporozoites within the skin. Strikingly, mosquitoes deposited fewer sporozoites in mice that had been actively or passively immunized against sporozoites. Similar results were obtained when immobilized mosquitoes were allowed to release saliva and sporozoites into drops of media on microscope slides. We provide evidence that these results are due to formation of an immune complex that reduces release of some sporozoites from the tip of the mosquito proboscis. Those sporozoites that were successfully injected into immunized mice were rapidly immobilized, did not appear to invade dermal blood vessels and became morphologically degraded within several hours.

## Results

### Mosquito Feedings on Ear Pinnae

**Figure 1:**
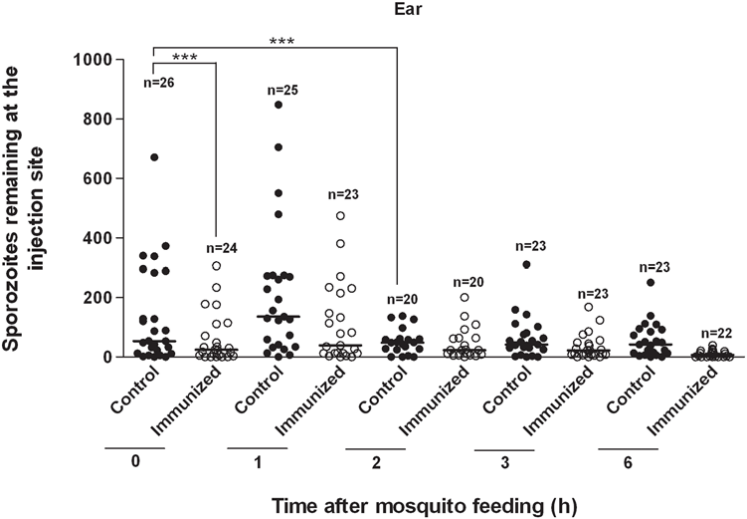
Mosquito injection of *Plasmodium berghei* sporozoites into mouse ear pinna. Scatter plot shows numbers of sporozoites remaining at bite site on ear at various times following injection of sporozoites by mosquitoes feeding on non-immunized (control) mice vs. mice actively immunized against sporozoites. Each point shows number of sporozoites left by a single mosquito. (N = total number of mosquito feedings for each time point.) Horizontal bars show medians. After data were log-transformed (see Materials and Methods), a 3-way analysis of variance (ANOVA) showed that the median for control mice at 2 h was significantly reduced (***P = 0.001) compared with 0-time mean; these reduced numbers stabilized at 3 and 6 h. The median number of sporozoites deposited in actively immunized mice at 0-time was reduced by greater than 45% compared with control mice at 0-time (***P<0.001). Counts of sporozoites injected into immunized mice were unreliable after 2 h due to deterioration and fragmentation of sporozoites.

A summary of numbers of sporozoites visualized at the bite site on the ear pinna after feedings by individual mosquitoes is presented as a scatter plot in **Fig. 1**. After mosquitoes fed on non-immunized (control) mice, we found a median of 53.5 sporozoites in biopsy specimens taken from the bite site immediately after feeding. We observed no significant differences in the numbers of these sporozoites compared with numbers found in biopsy specimens taken 1 h post feeding. Indeed, contrary to expectations, the median was greater at 1 h (median = 136); this was due to several high “outliers” observed in this 1 h group, resulting in an upwards skewing of the median. Thus, the variance within each of the groups was too great to permit detection of any significant differences between the medians for 0 vs. 1 h. However, there was a greater than 57% reduction in the number of sporozoites found 2 h post-feeding, compared with 0 h; this was highly significant; P = 0.001. Numbers of sporozoites remaining at the bite site after 2 h appeared to stabilize. In vivo gliding motility of sporozoites was high at 0 and 1 h, with a reduction observed at 2 h; no motility seen at 3 h and beyond. This was observed in mice that had been examined only once at either 1, 2, 3 or 6 h; thus the reduction in sporozoite motility over time was not due to sporozoite damage caused by excessive illumination during fluorescence microscopy.

**Figure 2:**
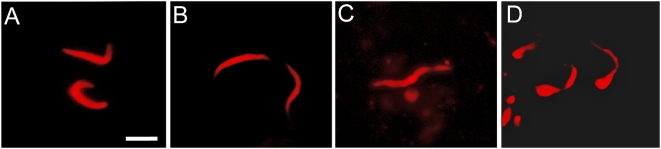
Confocal micrographs of remnant *Plasmodium berghei* sporozoites in the skin of control vs. immunized mice. A & B show typical presentation of sporozoites 2 and 4 h, respectively after mosquito inoculation into ear pinnae. C & D show typical presentation of sporozoites 2 and 4 h, respectively, after mosquito inoculation into ear pinna of actively immunized mice. Bar = 5 µm.

Immunized mice at 0 h had a median number of sporozoites that was less than 45% of the median number deposited in non-immunized 0 h controls; this reduction was highly significant; P<0.001. Motility of sporozoites in immunized mice ceased within min after deposition by mosquitoes. Counts of sporozoites injected into immunized mice were unreliable beyond 2 h due to deterioration and fragmentation of sporozoites (**Fig. 2**). Immunization status of mice was verified by ELISA, with use of a multiple antigen peptide specific for the *P. berghei* CSP for capture of anti-CSP antibodies in mouse sera. The geometric mean ELISA titer of sera taken from immunized mice on the day prior to challenge was 17,065 compared with <80 for sera from non-immunized controls. No immunized mice developed parasitemia after challenge by bite of individual mosquitoes, whereas 60% of the paired, non-immunized control mice developed parasitemia under the same conditions.

To test the correlation between numbers of sporozoites left at the bite site and whether the mice subsequently developed blood infections, we performed an additional study in which we allowed infected mosquitoes to bite non-immunized mice (1 mosquito per mouse) and assessed numbers of sporozoites within the ear at 1 h. We then followed the mice with daily Giemsa smears to assess patency. Seven of 18 mice (39%) developed blood infections, with a mean prepatent period of 6.7 days. The numbers of sporozoites found in 1 h biopsy specimens from these positive mice (median = 253) was significantly higher than the numbers of sporozoites found in 1-h biopsy specimens from the negative mice (median = 11); P = 0.0001.

### Mosquito Feedings on Abdomen

**Figure 3:**
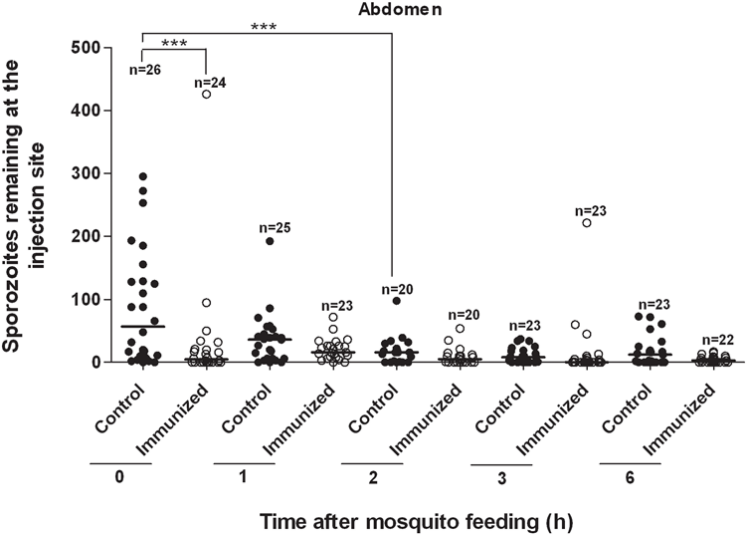
Mosquito injection of *Plasmodium berghei* sporozoites into mouse ventral abdomen. Scatter plot shows numbers of sporozoites remaining at bite site in ventral abdomen at various times following injection of sporozoites by mosquitoes feeding on non-immunized (control) mice vs. mice actively immunized against sporozoites. Each point shows number of sporozoites left by a single mosquito (N = total number of mosquito feedings for each time point). Horizontal bars show medians. Sporozoites in control mice at 1 h showed a 37% reduction compared to 0-time (not statistically significant), whereas there was a 72% reduction at 2 h, compared with 0-time (***P = 0.001). Numbers of sporozoites remaining at the bite site at 3 and 6 h appeared to stabilize, with no significant differences in the median number of sporozoites beyond the median numbers seen in the 2 h biopsy specimens. Significantly fewer sporozoites (***P<0.001) were deposited in immunized compared with control mice at 0 time. Counts of sporozoites injected into immunized mice were unreliable after 2 h due to deterioration and fragmentation of sporozoites.

A summary of numbers of sporozoites visualized at the bite site on the ventral abdomen (sum of the sporozoites found in the skin plus underlying tissues) after feedings by individual mosquitoes is presented as a scatter plot in **Fig. 3**. After mosquitoes fed on non-immunized (control) mice, we found a median of 57 sporozoites in biopsy specimens taken immediately after feeding. At 1 h there was a 37% reduction in numbers of sporozoites found in biopsy specimens but this reduction was not statistically significant. The numbers of sporozoites remaining at the bite site at 2 h (median = 16) was reduced by 72% compared with 0-time; P = 0.001. Numbers of sporozoites remaining at the bite site at 3 and 6 h appeared to stabilize, with no significant differences in the median number of sporozoites beyond the numbers seen in the 2 h biopsy specimens. As with the ear pinnae, in vivo gliding motility of sporozoites in abdominal skin was high at 0 and 1 h, with a reduction observed at 2 h and no motility seen at 3 h and beyond.

Actively immunized mice (status verified by ELISA) at 0-time had a 92% reduction in numbers of sporozoites deposited within abdominal tissues compared with their non-immunized 0-time control counterparts; P<0.001. Numbers of sporozoites seen in abdominal biopsy specimens from immunized mice seemed to stabilize beyond 0-time. As with the ear, however, counts of sporozoites injected into immunized mice were unreliable beyond 2 h due to deterioration and fragmentation of sporozoites. Motility of sporozoites within the abdominal skin of immunized mice ceased within min after deposition by mosquitoes.

### Mosquito Feedings after Passive Transfer of Monoclonal Antibodies

**Figure 4:**
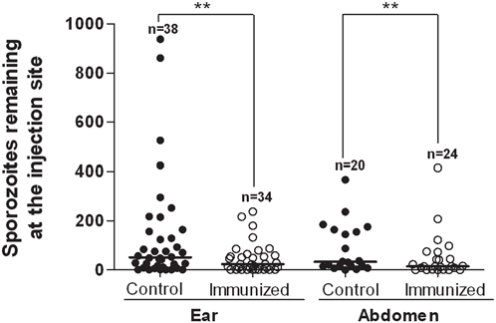
Mosquito injection of *Plasmodium berghei* sporozoites into mouse ear pinna or ventral abdomen (controls vs. mice passively immunized with anti-sporozoite antibodies). Scatter plot shows numbers of sporozoites deposited at bite site from biopsy specimens taken immediately after mosquito feeding. Each point shows number of sporozoites left by a single mosquito. (N = total number of mosquito feedings for each time point.) After mosquitoes fed on the ear pinnae of control mice, we found a median of 51 sporozoites deposited in the tissue; there was a 54% reduction in numbers of sporozoites injected into passively immunized mice (**P = 0.005). (IV-injected monoclonal antibodies were directed against repeat region of CSP of *P. berghei* sporozoites.) After mosquitoes fed on the ventral abdomen of control mice, we found a median of 33 sporozoites deposited in abdominal tissues; there was a 54.5% reduction in numbers of sporozoites injected into passively immunized mice (**P = 0.005).

To assess whether the reduced number of sporozoites deposited in actively immunized mice could be attributed to antibodies alone, we repeated the above studies with mice that had received passive IV transfer of a monoclonal antibody (MoAb 3D11), which is directed against the repeat region of the CS protein of *P. berghei* sporozoites. Control mice received either PBS or no injection. Challenge by bite of infected mosquitoes was delayed for 24 h to allow the MoAb to permeate avascular skin tissue. A summary of numbers of sporozoites visualized at the bite sites after feedings by individual mosquitoes is presented as a scatter plot in **Fig. 4**. After mosquitoes fed on the ear pinnae of control mice, we found a median of 51 sporozoites in biopsy specimens taken immediately after feeding; there was a 54% reduction in numbers of sporozoites injected into passively immunized mice; P = 0.005. After mosquitoes fed on the ventral abdomen of control mice, we found a median of 33 sporozoites in biopsy specimens taken immediately after feeding; there was a 54.5% reduction in numbers of sporozoites injected into passively immunized mice; P = 0.005. No immunized mice developed parasitemia after challenge by bite of individual mosquitoes, whereas 39% of the paired, non-immunized control mice developed parasitemia under the same conditions. As an additional negative control we passively immunized some mice in the same manner with MoAb NYS1, which is directed against the repeat region of the CS protein of *P. yoelii* sporozoites [9] and challenged these mice by bite of mosquitoes infected with *P. berghei* sporozoites (N = 8, with 2 ear bite sites and 2 abdominal bite sites for each mouse). There was no significant difference in numbers of sporozoites deposited in the ear pinnae or abdominal tissues of mice passively immunized with this heterologous antibody vs. non-immunized control mice, as determined by ANOVA (P>0.2).

### Intravital Microscopy Demonstrates Apparent Immune Complex Formed between CS Protein in Injected Mosquito Saliva and Anti-CS Antibodies in Mouse Tissue

**Figure 5:**
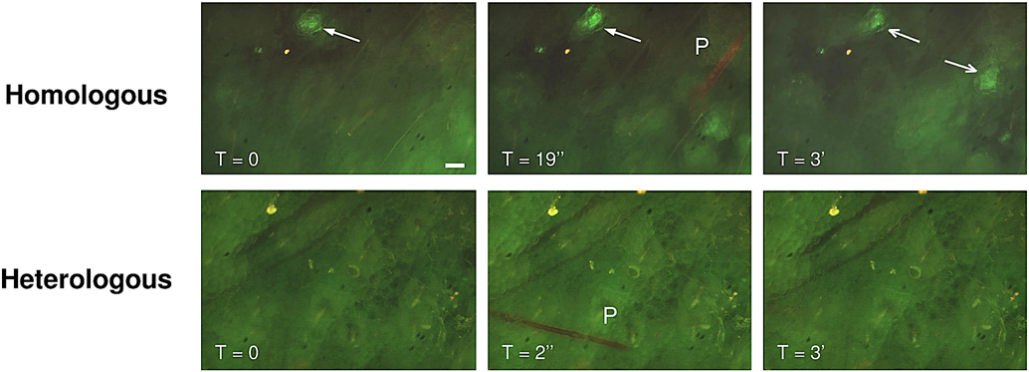
Intravital fluorescence micrographs showing proboscis introducing saliva into mouse ear pinnae after passive immunization with antibodies. Antibodies directed against repeat region of *Plasmodium berghei* CS protein had been conjugated with FITC prior to IV injection into mice. Upper Panel: Mosquitoes with salivary gland infection of *P. berghei* sporozoites injecting saliva that contains sporozoites in addition to secreted, soluble *P. berghei* CS protein. Green density (arrow at Time-0) shows apparent immune complex formed by interaction between soluble CS protein and homologous antibodies. (Proboscis had been observed at this site just prior to initiation of time-lapse video.) Bar = 100 µm for all frames. Probing proboscis (P) is visualized as orange due to autofluorescence, and was observed at 19 sec. At 3 min, apparent immune complex (right arrow) reached full intensity at site of mosquito probing. Micrographs are individual frames of 1 sec duration from Video S1. Lower Panel: Mosquito with salivary gland infection of *P. yoelii* sporozoites injecting saliva that contains sporozoites in addition to secreted, soluble *P. yoelii* CS protein. No interaction can be seen between soluble CS protein and the heterologous antibodies at sites of probing by proboscis (P). Micrographs are individual frames of 1 sec duration from Video S2. Similar negative results were observed with non-infected mosquitoes (Video S3).

Mice received an intravenous injection of FITC-conjugated MoAb 3D11and were challenged by mosquito bite 24 h later. When fed upon by mosquitoes with wild-type non-fluorescent *P. berghei* sporozoites (with soluble *P. berghei* CS protein in their saliva), green densities were visualized in mouse tissue close to the tip of the proboscis (**Fig. 5**, **upper panel** and **Video S1**). This was interpreted as formation of an immune complex (IC) at the distal end of the proboscis. When mice were fed upon by mosquitoes with *P. yoelii* sporozoites (with soluble *P. yoelii* CS protein in their saliva), no such green density could be observed as a result of contact with the heterologous anti-*P. berghei* antibodies (**Fig. 5**, **lower panel** and **Video S2**). Similarly, when mice were fed upon by non-infected mosquitoes, no green density could be observed in these negative controls (**Video S3**).

**Figure 6:**
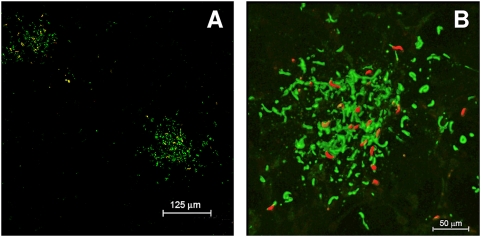
Fluorescence micrographs showing formation of apparent immune complexes after mosquito introduction of saliva and sporozoites into ear pinna of mouse that had been passively immunized with MoAb 3D11. Following mosquito bite, biopsy specimen of ear was fixed with ice-cold acetone and probed with fluorescein-conjugated Protein A, which specifically binds to the Fc component of antibodies. Fig. 6A shows that green staining of Protein A conjugate was restricted to sites where the proboscis had deposited red-fluorescent sporozoites. Fig. 6B is a higher power confocal micrograph showing a 3D reconstruction after the image was processed with Imaris 6.1.5 Bit Plane. The conjugate binds both to Fc portion of antibody attached to sporozoites, and to precipitated material between sporozoites in the vicinity of mosquito probes. No staining by the Protein A conjugate was seen in non-immunized mice challenged with *P. berghei* sporozoites or in mice passively immunized with 3D11 but challenged with heterologous *P. yoelii* sporozoites.

To further assess the nature of these green densities, we took biopsy specimens of the ear, and probed these with FITC-conjugated Protein A or Protein A/G. The results (**Fig. 6**) showed that these conjugates were specifically observed only in areas in which saliva and sporozoites had been deposited and only when the homologous combination of *P. berghei* sporozoites and 3D11 had been used, thus indicating the IC nature of these densities. No such focal FITC staining was seen in biopsy specimens from mice that had not been passively immunized with 3D11 or from passively immunized mice that had been challenged with heterologous *P. yoelii* sporozoites.

### Secretion of Mosquito Saliva into Medium in Vitro Demonstrates Antibody-Associated Blockage of Sporozoite Release

**Figure 7:**
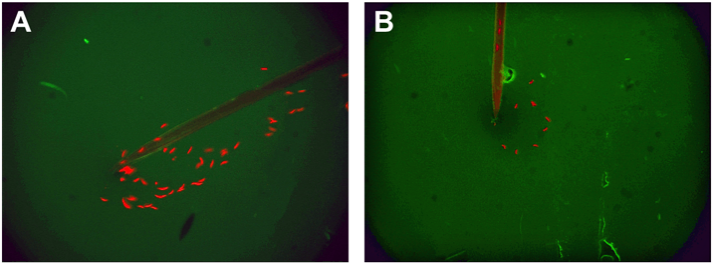
Fluorescence micrographs showing release of sporozoites from proboscis in drops of media on a glass slide. Fig. 7A shows free release of *Plasmodium berghei* sporozoites into medium containing BSA conjugated with FITC. Fig. 7B shows release of smaller numbers of sporozoites into medium containing MoAb 3D11(directed against *P. berghei*) conjugated with FITC. Sporozoites travelled more slowly down the proboscis and 3 sporozoites can be seen trapped within the lumen of the salivary duct within the proboscis. Micrographs are individual frames of 1 sec duration from Videos S4 and S5.

To further assess the nature of the apparent obstruction of sporozoite release by mosquitoes into a milieu of homologous antibodies, we did additional studies in which individual mosquitoes infected with fluorescent *P. berghei* sporozoites were immobilized on glass slides and we documented direct release of saliva and sporozoites into drops of medium containing either FITC-conjugated 3D11 or FITC-conjugated BSA (negative control). Sporozoites were consistently released freely from the distal end of the proboscis into control medium containing FITC-conjugated BSA (**Fig. 7A** and **Video S4**). When the infected mosquitoes were allowed to release saliva into the same medium containing FITC-conjugated 3D11, we observed that some of this medium was sucked back into the distal end of the proboscis and there was a relative stasis both of sporozoite transit through the salivary duct and release into the medium (**Fig. 7B** and **Videos S5** and **S6**). We made counts of sporozoites released into these media by individual mosquitoes (n = 20 mosquitoes). Because the data were not normally distributed, they were log transformed and an unpaired t-test was performed. The median number of sporozoites found was 86 in the BSA-containing control medium, and 21 in the MoAb 3D11-containing medium (P<0.01).

## Discussion

These results have confirmed previous findings that in addition to those sporozoites that move from the skin into the blood within minutes after deposition by mosquitoes [1], substantially more sporozoites take a significantly longer time to enter the blood, remaining, instead, in the skin for long periods of time [3],[5],[6]. In the current study, when we allowed infected mosquitoes to feed on the ear pinna of mice for 3 min, we were unable to detect any significant differences in numbers of sporozoites found within skin biopsy specimens taken immediately after feeding vs. 1 h post-feeding. Our earlier studies had shown that some *P. yoelii* sporozoites deposited into the ear pinna by mosquitoes begin to move into the blood within 15 min [1]. For this, we used a highly sensitive detection method (development of parasitemia) that can detect even a single sporozoite leaving the skin and subsequently traveling through the blood to infect the liver and initiate a blood infection; thus, this previous study was able to detect the earliest emigrants from the skin. Furthermore, intravital microscopy studies have clearly shown that some sporozoites move from avascular tissue to enter the blood circulation during the first hour after deposition by mosquitoes [3],[5].

Yet, our current study was unable to find any statistical difference between numbers of sporozoites found in the ear pinna at 0 vs. 1 h after feeding. These results imply that the numbers of sporozoites leaving the ear pinna within 1 h was relatively small and was undetectable within the background of the high variance we observed in numbers of sporozoites injected by individual, infected mosquitoes (**Fig. 1**: 0 vs. 1 h controls). When we evaluated the numbers of sporozoites remaining in the skin at later times after feeding, we found a significant loss of 57% of these late emigrants by 2 h compared with 0-time (*P*<0.001). Ultimately, there was a halt to this emigration, and we found a stabilization of sporozoite numbers within the ear in subsequent observations made beyond 2 h post-feeding. This correlates with our intravital microscopy observations of sporozoites within the ear pinna, which showed that sporozoite motility in the skin ceases within 2–3 h after mosquito inoculation of sporozoites. Amino *et al.* [5], who also performed a microscopic assessment of fluorescent *P. berghei* sporozoites injected into the ear pinna by individual mosquitoes, reported that ∼50% of the inoculated sporozoites left an observed portion of the bite site to invade either blood or lymphatic vessels within 1 h. Our observations that on the order of half the inoculated sporozoites ultimately remain in the skin of the ear pinna are similar to those reported by these authors [5], although differences in the rate of sporozoite departure from this site likely reflect differences in our methodologies. Both reports serve to quantify our earlier qualitative observations that large numbers of sporozoites remain in the skin after injection by mosquitoes [3].

Our comparative studies with individual mosquitoes feeding on the ventral abdomen showed substantially fewer sporozoites initially deposited per mosquito compared with numbers deposited in the ear, just as we had previously reported [8]. As we had concluded, this is likely the result of greater numbers of sporozoites being re-ingested by feeding mosquitoes, associated with more blood being re-ingested by mosquitoes feeding on the more heavily vascularized abdominal tissue [8]. Furthermore, mosquitoes feeding on heavily vascularized skin require fewer probes before they make contact with a source of blood; thus, less saliva and fewer sporozoites are likely to be delivered during this reduced probing time. In the current study, we found the numbers of sporozoites in biopsy specimens of abdominal tissues to be reduced by 37% at 1 h compared with 0-time specimens but this was not statistically significant. When we evaluated the numbers of sporozoites remaining in abdominal tissues at later times, we found a significant loss of 72% of sporozoites by 2 h compared with 0-time (P<0.001). There was a stabilization of sporozoite numbers within abdominal tissues in subsequent observations made beyond 2 h post-feeding. Our results differ considerably from those of Yamauchi *et al.* ([6], **Fig. 2C**) with *P. yoelii*, who reported that only on the order of 5% of sporozoites injected by mosquitoes into the back left the site within 1 h and were demonstrable as liver-stage exoerythrocytic forms (EEF). Whether this was due to differences in species or in technique or due to the fact that we measured loss of sporozoites from the skin, whereas Yamauchi *et al.* [6] counted only those sporozoites that transformed into EEF remains to be determined.

Our overall results for ear and abdomen showed that emigration of mosquito-deposited sporozoites was restricted largely to a 2 h period that correlated with the ability of these sporozoites to move within the skin; sporozoites that have not left the skin within this period appear destined to remain there. These results differ from those reported by Yamauchi *et al.* [6], who stated that, “infective sporozoites inoculated by the mosquito are released from the skin into the blood circulation in a trickle extending for hours after the mosquito bite.” Most of the experiments reported by these researchers were done with syringe-injected sporozoites, so they are not directly comparable to our own studies, which restricted itself to analysis of the kinetics of sporozoites injected by mosquitoes.

It has been well established that not all mosquitoes with salivary gland infections successfully transmit malaria to mammalian hosts [10]. In a previous study, we showed that some of this could be explained by a failure of ∼10% of the infected mosquitoes to transmit any sporozoites into the skin [8]. We now show that even mosquitoes injecting relatively few sporozoites into the skin are significantly more likely to fail to induce a blood infection. Mice that developed parasitemia subsequent to a bite on the ear pinna by individual mosquitoes had a median of 253 sporozoites found in the skin, whereas mice that failed to develop parasitemia had a median of only 11 sporozoites delivered (P = 0.0001). Because the biopsy specimens were taken 1 h after the bite, it is likely that some sporozoites had already left the bite site by this time; thus, numbers of sporozoites actually delivered had obviously been higher at 0-time. Nevertheless, we show elsewhere in our study that numbers of sporozoites leaving the ear within 1 h after the mosquito bite were relatively low. That mosquitoes delivering small numbers of sporozoites tend not to induce blood infections may be due to the relatively large percentage of sporozoites that never leave the skin. Mosquito-delivered sporozoites may either remain in the skin or exit it via dermal blood vessels [3],[5] or lymph vessels [5]. If comparatively few sporozoites are delivered into the skin in the first place, there is a greater chance that none will be successful in reaching the liver to continue the cycle leading to parasitemia.

Sporozoites injected by mosquitoes into mice that had been actively or passively immunized against sporozoites rapidly lost their motility as shown by intravital microscopy, as previously observed [3]. Because none of these mice developed a patent blood infection, it is presumed that none of the sporozoites successfully reached the liver and invaded hepatocytes from the blood. Our observation that fewer *P. berghei* sporozoites were deposited by mosquitoes into mice actively or passively immunized against *P. berghei* was unexpected. This was not seen when mice had been passively immunized with a heterologous MoAb directed against the repeat region of the *P. yoelii* CS protein. The simplest hypothesis to explain this phenomenon is that sporozoites were partially obstructed from leaving the proboscis by an immune complex formed by soluble CS protein released by sporozoites into the saliva [11]–[13] interacting with homologous anti-CS protein antibodies at the bite site. In support of this hypothesis, we observed an apparent precipitant reaction at the distal end of the proboscis during intravital fluorescence microscopy of *P. berghei*-infected mosquitoes feeding upon mice that had been passively immunized with antibodies against *P. berghei* sporozoites; these reaction sites were associated with specific binding of Protein A or A/G to sporozoites and to precipitated matter in the immediate vicinity of mosquito-deposited sporozoites, further implying the presence of IC at these sites [14],[15]. No such precipitant reactions or specific binding of Protein A or A/G was observed when we tested non-infected mosquitoes or mosquitoes infected with heterologous *P. yoelii* sporozoites.

Additional evidence for interference with mosquito delivery of sporozoites by homologous antibodies at the tip of the proboscis was furnished by studies in which we directly observed sporozoites being ejected from the proboscis into a drop of medium containing FITC-conjugated BSA or 3D11. When the fluid contained antibodies directed against homologous sporozoites, there was clear interference with ejection of the sporozoites, and significantly fewer were delivered into the drops of medium.

It has been recognized that pre-erythrocytic immunity can act at different times and sites: by sporozoite-immobilizing antibodies in avascular tissue that block sporozoites from reaching blood vessels that can carry them to the liver [3], by antibodies in the circulation that may prevent IV-injected sporozoites from invading the liver [16] and by CD4+ and CD8+ T-cells that can act against infected hepatocytes [17],[18]. We now propose an additional immune effector mechanism, namely, that mosquitoes inject significantly fewer sporozoites into immunized hosts in the first place. The flow of sporozoites released in saliva from the proboscis is already restricted to a limited number of sporozoites under normal circumstances [8],[19]; further restriction by release of this saliva into an obstacle formed by an immune complex precipitate could additionally reduce the number of injected sporozoites, thus contributing to the protective effect of pre-erythrocytic immunity. Whether this also occurs in semi-immune humans in an endemic area remains to be determined. It has long been recognized that the size of a sporozoite inoculum may influence the nature of the subsequent blood infection in human malaria (rev. in [20]). Thus, an antibody-mediated reduction in numbers of sporozoites injected into semi-immune humans might play a role in limiting the intensity of the ensuing blood infection in combination with a subsequent immune response against blood stages of the parasite.

## Materials and Methods

### Sporozoites

*Anopheles stephensi* mosquitoes were infected with a clone of the rodent malaria parasite, *P. berghei*, whose sporozoites constitutively express RedStar, an improved red fluorescent protein [21]. For some studies we used mosquitoes infected with wild-type *P. berghei* (strain NK65) or *P. yoelii* (strain 17NXL), neither of whose sporozoites express fluorescent protein. We used standard protocols for infecting and maintaining mosquitoes [22], which were infected by feeding upon gametocyte-carrying 6–8 wk-old Swiss-Webster mice (Taconic Farms Inc., Germantown, NY). Our protocols for maintenance and use of experimental animals were approved by the Institutional Animal Care and Use Committee at New York University School of Medicine, and our animal facility is accredited by the Association for Assessment and Accreditation of Laboratory Animal Care International (Rockville, MD). Mosquitoes were used for sporozoite transmission studies 18 days after the infective blood meal. Prior to use of infected mosquitoes for feedings observed by intravital microscopy, live, intact mosquitoes were examined by fluorescence microscopy to establish that they had salivary gland infections [23],[24]; mosquitoes found to be negative were discarded.

### Mosquito Feeding on Ear Pinnae

For these and other sporozoite transmission studies, mosquitoes fed on BALB/c mice anesthetized by IP injection of ketamine (50 mg/kg) plus xylazine (10 mg/kg) and acepromazine (1.7 mg/kg), and placed on a warming tray. To restrict the area of sporozoite deposition for more efficient counting of sporozoites, the dorsal aspect of one ear pinna was partially masked with tape so that only its edge (8–10 mm long and 2–3 mm wide) was accessible to a feeding mosquito. Mosquitoes, previously selected for having positive salivary gland infections, were kept individually in plastic feeding tubes 2.5 cm in length and with an inside diameter of 1.5 cm; one end of the tube was covered with netting through which the mosquito was able to feed and the other end was closed with a screw-cap. Each mosquito was allowed to probe and feed on the ear through the netting for 3 min from the time that probing was first observed.

At appropriate times after each feeding, the fed-upon region of the ear plus the taped adjacent area ∼2.5 mm beyond this was excised. This biopsy specimen was separated into dorsal and ventral leaflets with fine forceps [25], after which each leaflet was mounted under a coverslip and examined by fluorescence microscopy to count sporozoites and record their distribution [8]. Biopsy specimens were taken either immediately after feeding or at intervals of 1, 2, 3 or 6 h after feeding. Parallel studies were done with mice that had been actively or passively immunized against sporozoites. Fed-upon mice were kept for up to 14 days to obtain blood smears from the tip of the tail; smears were stained with Giemsa and observed by bright field microscopy to detect patent blood infections. This is an extremely sensitive way to establish whether even a single sporozoite has left the skin to develop further in the liver and establish a blood infection.

### Mosquito Feeding on Ventral Abdomen

Mosquitoes were allowed to feed on mice anesthetized as above. Hair was removed from an area of the ventral abdomen with a razor blade. Anesthetized mice were placed on a warming tray, ventral side facing up, and a portion of the abdominal skin was masked with tape that had a 4 mm-diameter hole punched into it. Mosquitoes placed individually in plastic feeding tubes, as above, were allowed to probe and feed through the hole in the tape for 3 min from the time that probing was first observed. Immediately after feeding, the periphery of the feeding circle was marked and the tape was removed. At appropriate times after each feeding, the full depth of skin of a circle 6 mm in diameter, centered around the bite site, was removed with a skin punch device (6 mm Miltex Biopsy Punch) while mice were under deep anesthesia. Then, an underlying circle of peritoneal muscle wall was removed and both incisions were closed [8]. Both biopsy specimens were mounted under cover-slips and viewed through a fluorescence stereoscopic microscope to count sporozoites, as above. We added the numbers found in the skin to the numbers found in peritoneal musculature to obtain the total numbers of sporozoites remaining in the ventral abdomen after mosquito feedings [8]. Biopsy specimens were taken either immediately after feeding or at intervals of 1, 2, 3 or 6 h after feeding. Parallel studies were done with mice that had been actively or passively immunized against sporozoites. For follow-up information, mice, were maintained for blood smears, as above, to detect patent blood infections.

### Immunization

Sporozoites for immunization were first purified on a DEAE ion-exchange column [26] in order to distinguish between effects due to anti-sporozoite vs. anti-saliva immunity. Purified sporozoites were irradiated within a gamma irradiator (MDS Nordion Gammacell® 1000 Elite) to a central dose of ±12049 cGy and a minimum dose of ±10266 cGy. Mice received an IV injection of 50,000 irradiated sporozoites, with 2 subsequent booster injections of 10,000 irradiated sporozoites, each, at 15 days intervals. They were challenged by mosquito bite 15 days after the second boost. Serum from immunized mice was taken the day prior to challenge to assess levels of anti-sporozoite antibodies.

Some mice were passively immunized by IV injection of 320 µg per mouse of MoAb 3D11, directed against the repeat region of *P. berghei* CS protein [3] or with MoAb NYS1, directed against the repeat region of *P. yoelii* CS protein [9]. All mice were challenged by bite of infected mosquitoes 24 h after antibody transfer. Parallel challenges were done on non-immunized control mice.

### Visualisation of Immuno-Complexes Formation at the Injection Site

For these studies MoAb 3D11 was conjugated with FITC prior to injection. For this, 150 µl of 1 mg/ml FITC solution (Fluorescein Isothiocyanate; Sigma, St. Louis, MO) was added to a 2 mg/ml 3D11 solution (or BSA for negative controls) and incubated at room temperature for 1 h. Unbound FITC was then separated from the conjugate with a PD-10 gel filtration column loaded with Sephadex G-25 M (Amersham Biosciences, Piscataway, NJ, USA). Mice were passively immunized by IV-injection of 150 µg per mouse of the conjugates. On the next day, the ear pinnae of these mice were fed upon by mosquitoes infected with *P. berghei* or *P. yoelii* sporozoites (none of these sporozoites expressing fluorescent protein) or by non-infected mosquitoes, while intravital fluorescence videomicroscopy observations were made of the feedings, with particular focus on the site of injection of saliva from the distal end of the proboscis. Subsequent to these observations, biopsy specimens were taken from the fed-upon ears and probed with FITC-conjugated Protein A or Protein A/G (Pierce Biotechnology IL, USA; 20 µg/mL).

### Sporozoite Injection into Drops of Media

To study ejection of saliva and sporozoites by mosquitoes into media, we used a modification of the method of Frischknecht et al. (2004) [27]. The feeding stylets of individual mosquitoes immobilized on a microscope slide were positioned under a 13×13 mm coverslip and the mosquito was allowed to salivate into 5 µl of RPMI medium containing 1 mg/µl of either FITC-conjugated BSA (control) or FITC-conjugated MoAb 3D11. Salivation was observed for 10 min by videomicroscopy with the Leica MZ16FA fluorescence stereoscopic microscope, after which counts were made of the sporozoites released into the media.

### ELISA Assay

Antibody titers were determined by enzyme-linked immunosorbent assay (ELISA), using the *P. berghei*-specific B4 multiple Ag peptide (MAP) as antigen (4 branch MAPS, each branch with 3 repeats of DPPPPNPN) from AnaSpec, Inc., San Jose, CA) [28]. Peroxidase-labeled anti-mouse IgG was used as a secondary antibody and 2,2′-azinobis(3-ethylbenthiazolinesulfonic acid) (ABTS) as the substrate. The end point was measured as the highest dilution of serum having a delta O.D. greater than the mean+3 standard deviations obtained with non-immune sera. Results were expressed as geometric mean titers.

### Microscopy

For counting of sporozoites, we used a Leica MZ16FA fluorescence stereoscopic microscope with a 2.0× stereoscopic objective lens. Illumination for fluorescence studies was with an EXFO X-Cite 120 F1 illumination system and with a DsRED filter set, restricting illumination to 515–556 nm (peak = 545) and signal emission to 590 nm. Observations of remnant sporozoites and Protein A or A/G staining in the skin of control and immunized animals were performed using a Leica Inverted Laser Scanning Confocal Microscope (Model Number TCS SP2 AOBS) and Leica LCS Software. Each image is the average projection superimposed stacks of individual focal plane images. The total thickness of the stack varies depending upon the position of the sporozoites in the biopsy specimen. Intravital videomicroscopy was done with a Leica DMI 4000B inverted fluorescence microscope with a 10× objective lens. Illumination for fluorescence studies was with a CTR4000 illumination system and with a dual Green/Red filter set, restricting illumination to 480–500 nm (peak = 490) and 560–590 nm (peak = 575) and signal emission to 505 and 600 nm. Images were acquired with a Leica DFC300 FX digital camera and saved as digital files for further analysis and processing. We used Leica Application Suite software (LAS V2.7.1) for documentation and analysis. For 3D reconstruction and volume rendering, raw 3D data set were processed using Imaris 6.1.5 (BitPlane, 2008) software. A Gaussian filter was used for noise reduction on the average projection and Iso-surface objects were created on an intensity value on a per channel basis. Surfaces were colored in green for the green channel (FITC-conjugated Protein A) and red for the red channel (*P. berghei* Red Star). Green surfaces were attributed a 50% transparency in order to visualize double staining.

### Statistics

The numbers of sporozoites injected by mosquitoes did not follow a Normal distribution but were highly skewed with a clear floor effect. However, when log-transformed [ln (spz count+1)], the data sufficiently approximated a Normal distribution that allowed the use of parametric tests. Analysis of variance (ANOVA) examined the effects of site (abdomen vs. ear), immunization status (control vs. immunized) and time (0 to 6 h), as appropriate; Student's t-test (unpaired, 2-tailed) compared means when only two experimental groups were considered. The analyses were performed using SPSS 15.0 for Windows (SPSS Inc., 2006) and GraphPad Prism Version 5 software (San Diego, California.

## Supporting Information

Video S1 Intravital fluorescence video showing mosquito proboscis introducing saliva into mouse ear pinna after passive immunization with fluorescent antibodies (homologous antigen and antibodies). Mosquitoes had salivary gland infection of *Plasmodium berghei* sporozoites. Mouse had received passive IV transfer of anti-*P. berghei* sporozoite monoclonal antibodies conjugated with FITC. Saliva contained sporozoites in addition to secreted, soluble *P. berghei* CS protein. Green density (left arrow at start of video) shows apparent immune complex formed by interaction between soluble CS protein and homologous antibodies. (Proboscis had been observed at this site just prior to initiation of time-lapse video.) Another green density (right arrow) is seen resulting from probe of another proboscis. Proboscis is visualized as orange due to autofluorescence. During 3-min, real-time duration of video, several additional green densities are formed from probosci that are out of the field of focus. Because of background density caused by FITC-labeled antibodies in tissue, relatively long exposure times of 1 sec per frame were required. Bar = 100 µm for all videos.
(2.26 MB MOV)

Video S2 Intravital fluorescence video showing mosquito proboscis introducing saliva into mouse ear pinna after passive immunization with fluorescent antibodies (heterologous antigen and antibodies). Mosquitoes had salivary gland infection of *Plasmodium yoelii* sporozoites. Mouse had received passive IV transfer of anti-*P. berghei* sporozoite monoclonal antibodies conjugated with FITC. Saliva contained sporozoites in addition to secreted, soluble *P. yoelii* CS protein. Probing proboscis is visualized as orange due to autofluorescence. No proboscis-associated green densities were ever seen during our observations.
(1.30 MB MOV)

Video S3 Intravital fluorescence video showing mosquito proboscis introducing saliva into mouse ear pinna after passive immunization with antibodies (negative control). Mosquitoes had no salivary gland infections with sporozoites. Mouse had received passive IV transfer of anti-*P. berghei* sporozoite monoclonal antibodies conjugated with FITC. Saliva contained neither sporozoites nor sporozoite antigen. Probing proboscis is visualized as orange due to autofluorescence. No proboscis-associated green densities were ever seen during our observations, although secreted droplets of saliva (arrows) can be seen associated with two of the three probes within this sequence.
(2.31 MB MOV)

Video S4 Secretion of saliva and *P. berghei* sporozoites from proboscis of immobilized mosquito into medium on microscope slide: Medium contained FITC-conjugated BSA. Sporozoites were freely released into medium. Secretion of saliva can be seen as a dark globule at the tip of proboscis. Green streak running along middle of distal end of proboscis indicates that some medium was being sucked back by mosquito. (Real-time duration of video was 5 min, 32 sec; each frame was captured for 1 sec.)
(5.52 MB AVI)

Video S5 Secretion of saliva and *P. berghei* sporozoites from proboscis of immobilized mosquito into medium on microscope slide: Medium contained FITC-conjugated 3D11. Relatively few sporozoites were released into medium. Some of these have become coated with antibody and fluoresce green. Green plug at end of proboscis is associated with stasis of unreleased, red-fluorescing sporozoites within proboscis, suggesting formation of an immune complex that inhibits sporozoite release. (Real-time duration of Video S5 was 2 min, 22 sec; each frame was captured for 1 sec.)
(4.31 MB AVI)

Video S6 Secretion of saliva and *P. berghei* sporozoites from proboscis of immobilized mosquito into medium on microscope slide: Medium contained FITC-conjugated 3D11. Relatively few sporozoites were released into medium. Some of these have become coated with antibody and fluoresce green. Green plug at end of proboscis is associated with stasis of unreleased, red-fluorescing sporozoites within proboscis, suggesting formation of an immune complex that inhibits sporozoite release. (Real-time duration of Video S6 was 3 min, 28 sec; each frame was captured for 1 sec.)
(3.44 MB AVI)
